# The long non-coding RNA PTTG3P promotes growth and metastasis of cervical cancer through PTTG1

**DOI:** 10.18632/aging.101830

**Published:** 2019-03-10

**Authors:** Xiang-cui Guo, Li Li, Zhi-hui Gao, Hong-wei Zhou, Jun Li, Qian-qing Wang

**Affiliations:** 1 Gynecologic Oncology, Xinxiang City Central Hospital, Xinxiang 453000, Henan, China; 2 Nuclear Medicine Department, Xinxiang City Central Hospital, Xinxiang 453000, Henan, China

**Keywords:** cervical cancer, PTTG3P, Pituitary Tumor Transforming Gene 1 (PTTG1), cancer growth and metastasis

## Abstract

The outgrowth and metastasis of cervical cancer (CC) contribute to its malignancy. Pituitary Tumor Transforming Gene 1 (PTTG1) is upregulated in many types of cancer, and enhances tumor cell growth and metastasis. However, the activation and regulation of PTTG1 in CC, especially by its pseudogene PTTG3P, have not been shown. Here, we detected significantly higher levels of PTTG1 and PTTG3P in the resected CC tissue, compared to the paired adjacent normal cervical tissue. Interestingly, the PTTG3P levels positively correlated with the PTTG1 levels. High PTTG3P levels were associated with poor patients’ survival. In vitro, PTTG1 were increased by PTTG3P overexpression, but was inhibited by PTTG3P depletion in CC cells. However, PTTG3P levels were not altered by modulation of PTTG1 in CC cells, suggesting that PTTG3P is upstream of PTTG1. Moreover, PTTG3P increased CC cell growth, likely through CCNB1-mediated increase in cell proliferation, rather than through decrease in cell apoptosis. Furthermore, PTTG3P increased CC cell invasiveness, likely through upregulation of SNAIL and downregulation of E-cadherin. Our work thus suggests that PTTG3P may promote growth and metastasis of CC through PTTG1.

## Introduction

Cervical cancer (CC) typically occurs in the cervix as squamous cell carcinomas, largely resulted from sustained infections with human papillomavirus (HPV) [1–3]. The malignancy of CC is mainly contributable to its outgrowth and metastasis, which leads to a compelling requirement for a full comprehension of the molecular mechanisms controlling CC growth and invasiveness [1].

Pituitary Tumor Transforming Gene 1 (PTTG1) is a potent cell-cycle activator [4]. The encoded protein for PTTG1 is a homolog of yeast securin proteins, and functions through induction of sister chromatid separation [4]. It is a critical substrate that joins to an anaphase-promoting complex (APC) with separin to lead to the APC activation [4]. Interestingly, PTTG1 is highly expressed in many different types of tumors, and enhances tumor cell growth and metastasis through complicated while not-fully-determined mechanisms [5–11].

Pituitary tumor-transforming 3 (PTTG3P; NCBI Accession NO.NR_002734) [12], is a pseudogene highly homologous to its parental gene, PTTG1. PTTG3P has a limited protein-coding capacity, and thus is regarded as one long non-coding RNA (lncRNA) implied in tumorigenesis of gastric carcinoma [13] and hepatocellular carcinoma (HCC) [14]. Recently, lncRNAs have been found to essentially participate into the regulation of cellular development, differentiation, cell-fate determination and tumorigenesis [15–20]. Indeed, mutation and dysregulation of lncRNAs are found to be associated with cancer initiation, growth and metastasis [21–26]. However, reports on the function and clinical significance of CC-related lncRNAs are very rare [27]. Moreover, the activation and regulation of PTTG1 in CC, especially by its pseudogene PTTG3P, have not been shown.

Here, we studied the activation and regulation of PTTG1 in CC, and their association with the expression of the pseudogene of PTTG1, PTTG3P.

## RESULTS

### High PTTG1 and PTTG3P levels are detected and positively correlated in CC specimens

**Figure1:**
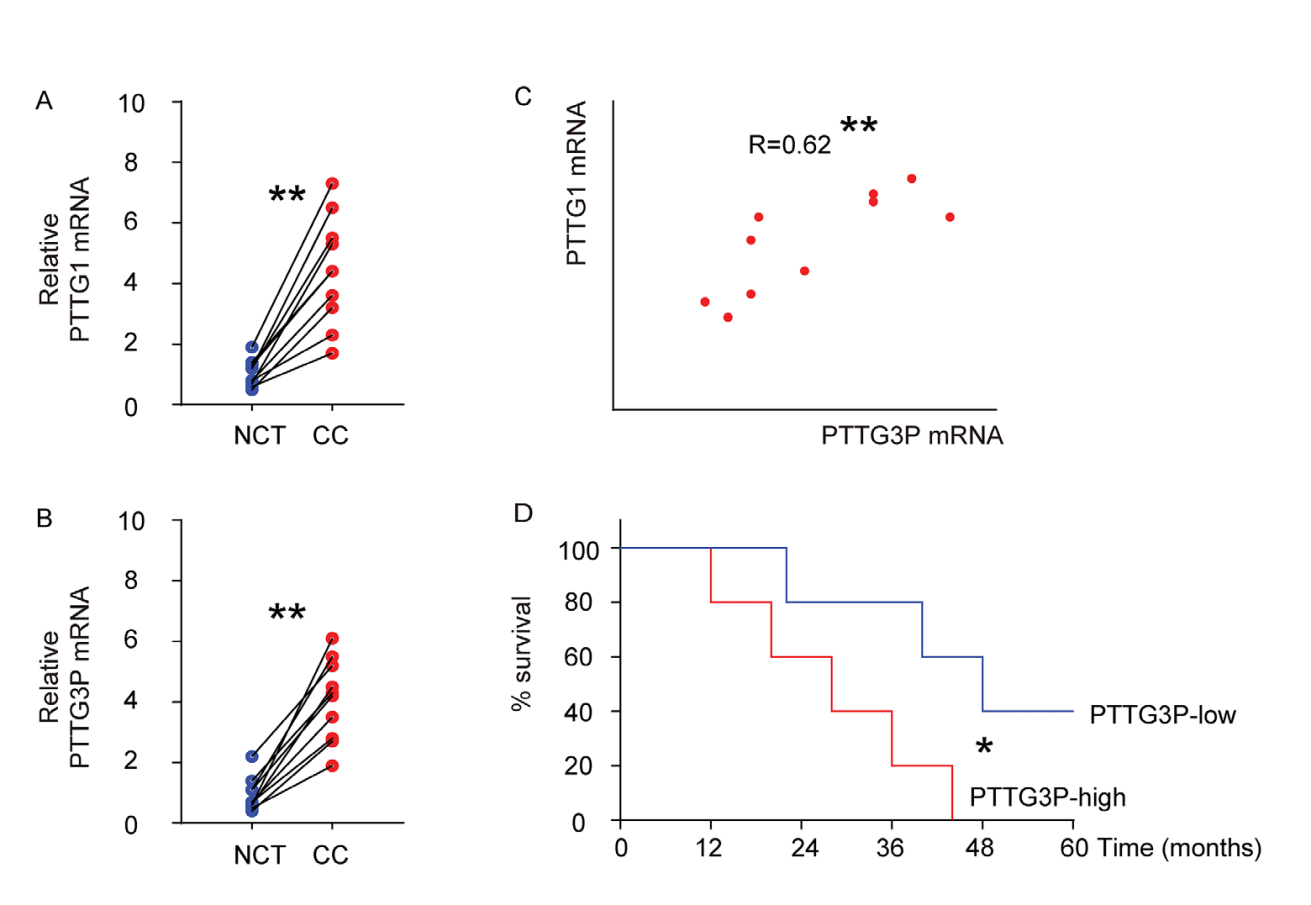
**High PTTG1 and PTTG3P levels are detected and positively correlated in CC specimens.** (**A-B**) The mRNA levels of PTTG1 (**A**) and PTTG3P (**B**) were examined in 10 CC specimens, compared to the paired adjacent normal cervical tissue (NCT) by RT-qPCR. Data were analyzed using Wilcoxon Test. (**C**) Bivariate correlations between PTTG1 and PTTG3P levels in CC were calculated by Spearman's rank correlation coefficients. (**D**) Kaplan-Meier curve was applied to record the overall survival of the patients that were differentiated into PTTG3P-high group and PTTG3P-low group using the cut-off point of median level of PTTG3P. *p<0.05. **p<0.01. N=10.

We examined the mRNA levels of PTTG1 and PTTG3P in 10 CC specimens, compared to the paired adjacent normal cervical tissue (NCT) by RT-qPCR (Figure 1A-B). We found that compared to NCT, CC tissue had a significantly higher level of PTTG1 (Figure 1A), and a significantly higher level of PTTG3P (Figure 1B). Moreover, we detected a strong and positive correlation between the levels of PTTG1 and PTTG3P in the all examined CC specimens (Figure 1C, R=0.62, p<0.01). The median value of PTTG3P in these patients was used as a cutoff point to separate the total samples into 5 PTTG3P-high group and 5 PTTG3P-low group. These 10 patients were followed up for 5 years. The Kaplan-Meier curves for the overall 5-year survival of these patients showed a poorer survival for those with higher PTTG3P (p<0.05; Figure 1D).

### PTTG3P is upstream of PTTG1 in CC

**Figure 2:**
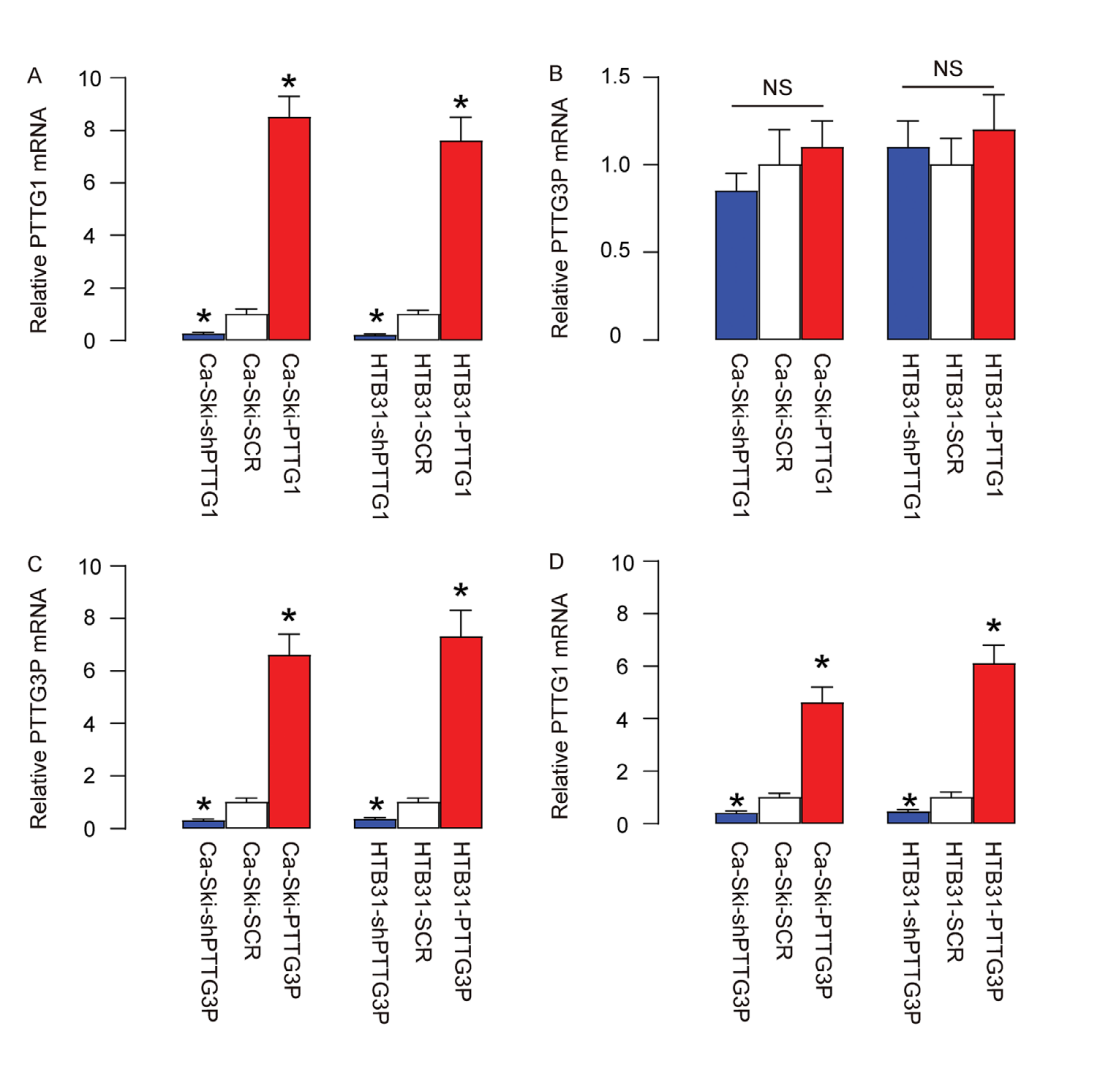
**PTTG3P is upstream of PTTG1 in CC.** (**A-B**) We overexpressed PTTG1 or depleted PTTG1 (by expressing short hairpin interfering RNA for PTTG1-shPTTG1) in two CC lines, Ca-Ski and HTB31. Plasmids carrying a scrambled sequence (SCR) were used as controls. (**A**) RT-qPCR for PTTG1. (**B**) RT-qPCR for PTTG3P. (**C-D**) We overexpressed PTTG3P or depleted PTTG3P (by expressing short hairpin interfering RNA for PTTG3P-shPTTG3P) in two CC lines, Ca-Ski and HTB31. (**C**) RT-qPCR for PTTG3P. (**D**) RT-qPCR for PTTG1. *p<0.05 (versus SCR). NS: non-significant. N=5.

In order to determine the regulatory relationship between PTTG1 and PTTG3P, we overexpressed PTTG1 or depleted PTTG1 (by expressing short hairpin interfering RNA for PTTG1-shPTTG1) in two CC lines, Ca-Ski and HTB31, or overexpressed PTTG3P or depleted PTTG3P (by expressing short hairpin interfering RNA for PTTG3P-shPTTG3P) in these two CC lines. Plasmids carrying a scrambled sequence (SCR) were used as controls. After confirmation of the effects of PTTG1 or shPTTG1 (Figure 2A), we found that modulation of PTTG1 levels did not alter PTTG3P levels in CC cells (Figure 2B). However, after confirmation of the effects of PTTG3P or shPTTG3P (Figure 2C), we found that overexpression of PTTG3P levels significantly increased PTTG1 levels in CC cells, while depletion of PTTG3P levels significantly decreased PTTG1 levels in CC cells (Figure 2D). These data suggest that PTTG3P is upstream of PTTG1 in CC.

### PTTG3P promotes CC growth through increase in cell proliferation

**Figure 3:**
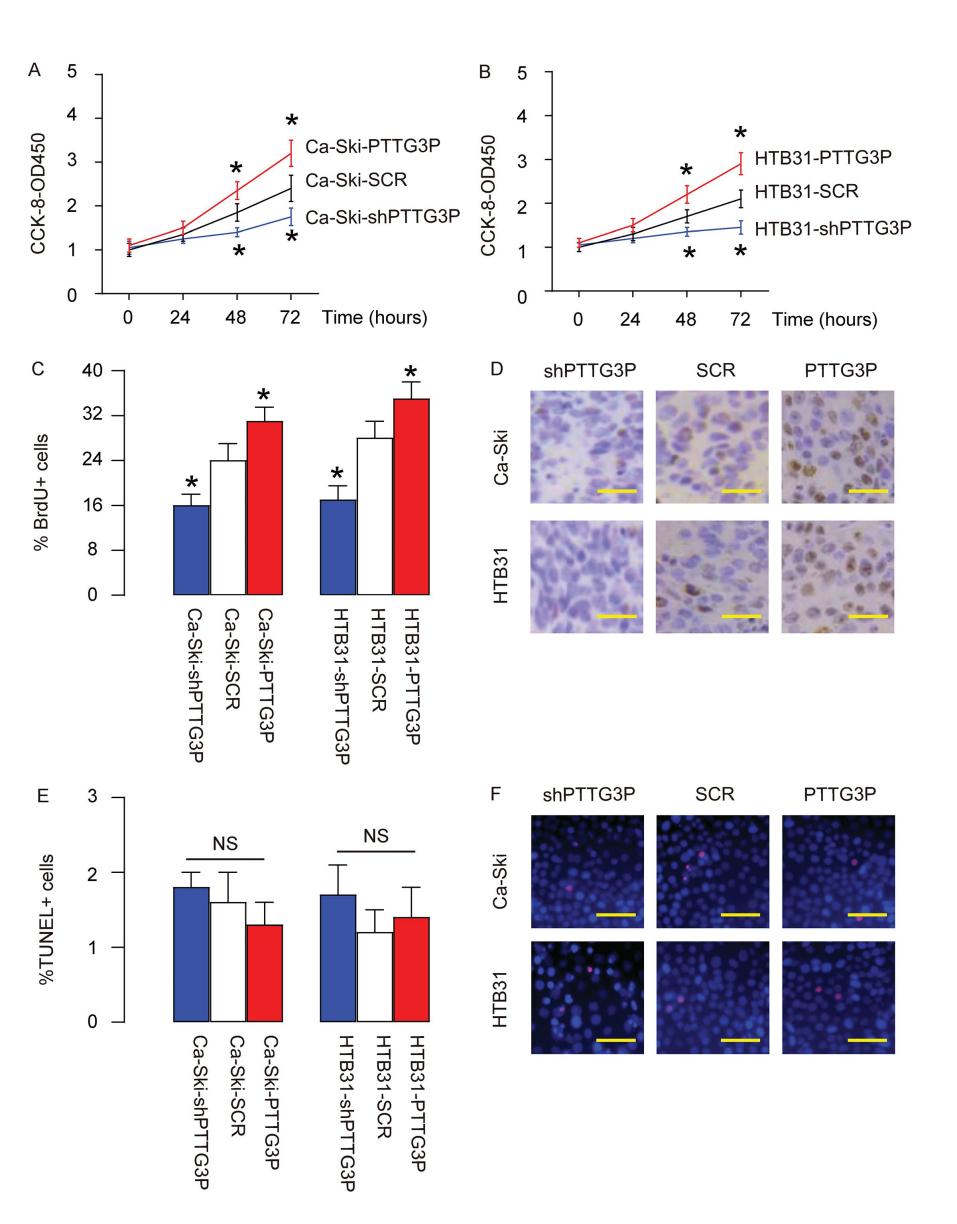
**PTTG3P promotes CC growth through increase in cell proliferation.** (**A-B**) The effects of PTTG3P on the growth of Ca-Ski cells (**A**) and HTB31 (**B**) were assessed in an CCK-8 assay. (**C-D**) BrdU assay was used to measure cell proliferation, shown by quantification (**C**), and by representative images (**D**). (**E-F**) TUNEL assay was used to measure cell apoptosis, shown by quantification (**E**), and by representative images (**F**). *p<0.05 (versus SCR). NS: non-significant. N=5. Scale bars are 20µm.

Next, effects of PTTG3P on the growth of CC cells were assessed. In an CCK-8 assay, we found that overexpression of PTTG3P significantly increased growth of Ca-Ski cells (Figure 3A) and growth of HTB31 cells (Figure 3B), while depletion of PTTG3P significantly decreased growth of Ca-Ski cells (Figure 3A) and growth of HTB31 cells (Figure 3B). BrdU assay showed that overexpression of PTTG3P significantly increased BrdU+ Ca-Ski cells and BrdU+ HTB31 cells, while depletion of PTTG3P significantly decreased BrdU+ Ca-Ski cells and BrdU+ HTB31 cells, shown by quantification (Figure 3C), and by representative images (Figure 3D). TUNEL assay showed that either overexpression of PTTG3P or depletion of PTTG3P did not significantly alter the percentage of TUNEL+ cells, shown by quantification (Figure 3E), and by representative images (Figure 3F). Together, these data suggest that PTTG3P may promote CC growth through increase in cell proliferation.

### PTTG3P increases CC invasiveness

**Figure 4:**
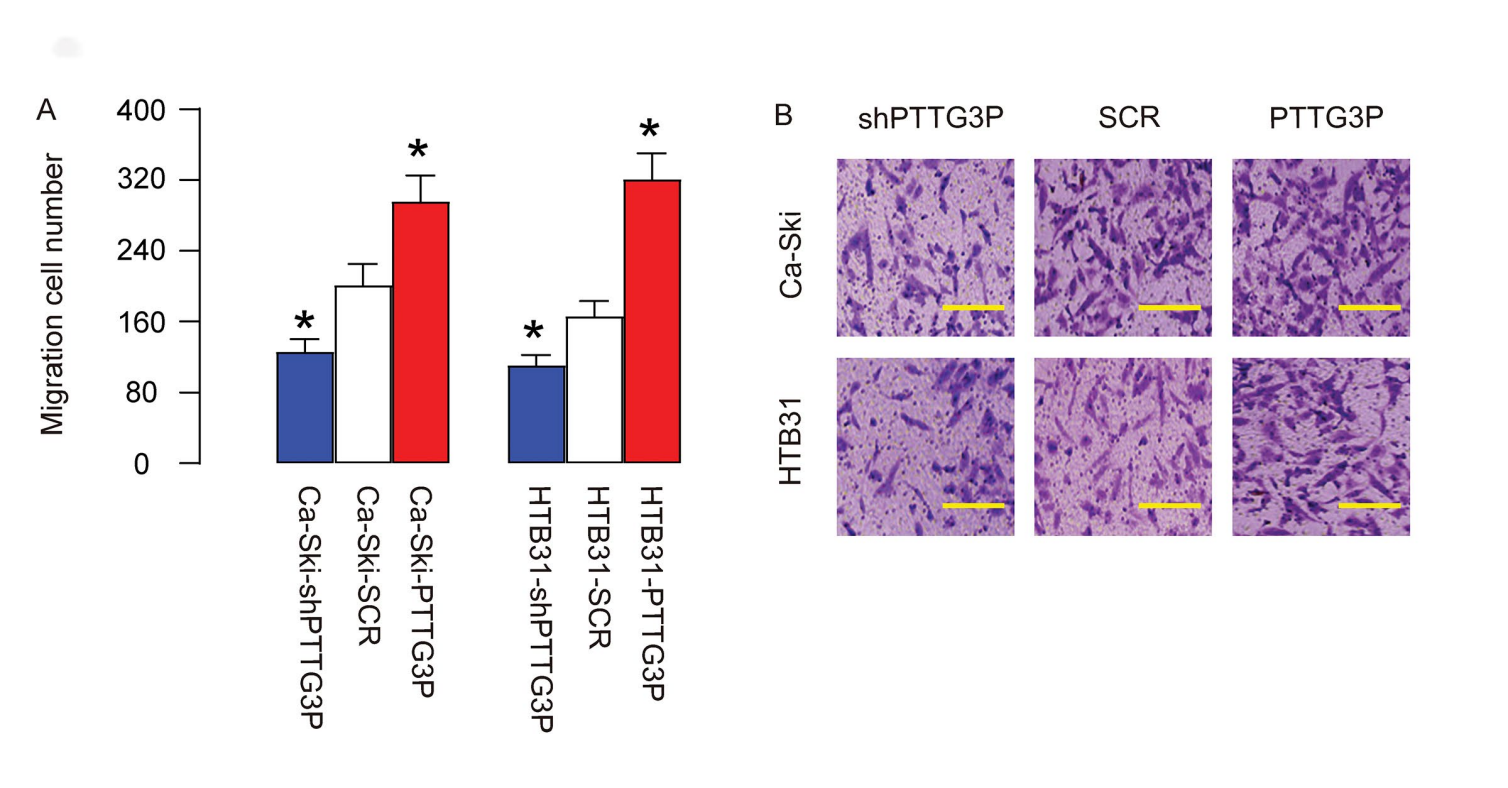
**PTTG3P increases CC invasiveness.** (**A-B**) The role of PTTG3P in CC cell invasiveness was assessed in a Transwell cell migration assay, shown by quantification (**A**), and by representative images (**B**). *p<0.05 (versus SCR). N=5. Scale bars are 30µm.

Then we examined the role of PTTG3P in CC cell invasiveness. We found that overexpression of PTTG3P significantly increased cell invasiveness in both Ca-Ski and HTB31 cells, while depletion of PTTG3P significantly decreased cell invasiveness in both Ca-Ski and HTB31 cells, in a Transwell cell migration assay, shown by quantification (Figure 4A), and by representative images (Figure 4B). Thus, PTTG3P increases CC invasiveness.

### PTTG3P increases CCNB1, SNAIL and inhibits E-Cadherin

**Figure 5:**
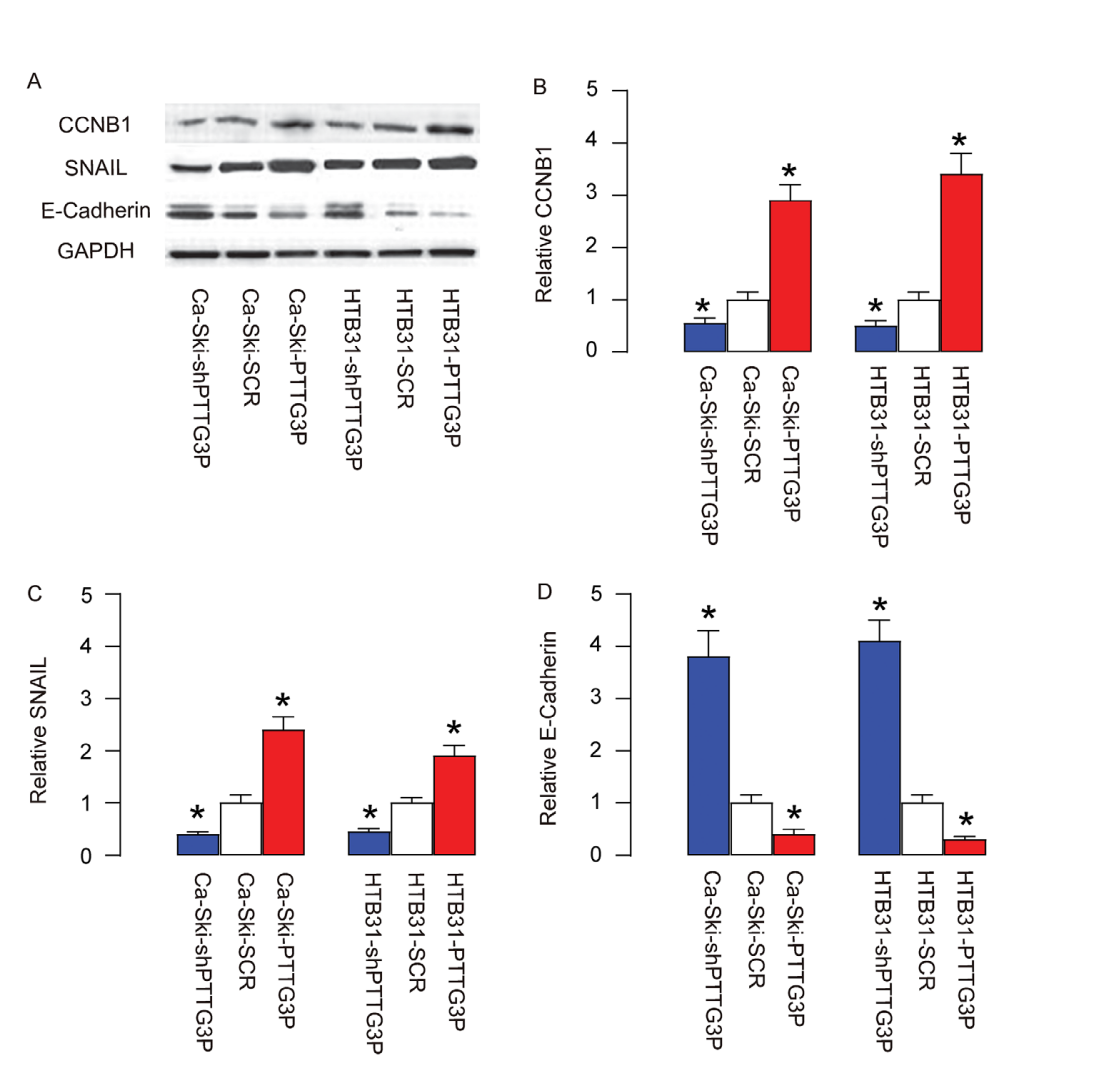
**PTTG3P increases CCNB1, SNAIL and inhibits E-Cadherin.** (**A**) Representative gels for Western blot. (**B**) Quantification for CCNB1. (**C**) Quantification for SNAIL. (**D**) Quantification for E-Cadherin. *p<0.05 (versus SCR). N=5.

In order to understand the molecular mechanisms underlying the effects of PTTG3P on cell growth and invasiveness, we analyzed the expression of factors controlling cell cycle and cell metastasis. Specifically, we found that PTTG3P increases CCNB1 (Figure 5A-B), SNAIL (Figure 5A, C) and inhibits E-Cadherin (Figure 5A, D). These data suggest that PTTG3P promotes CC growth, likely through CCNB1-mediated increase in cell proliferation. Moreover, PTTG3P increases CC cell invasiveness, likely through upregulation of SNAIL and downregulation of E-cadherin.

## DISCUSSION

The PTTG1 is highly expressed in some human cancers. Mechanistically, PTTG1 has been shown to induce cellular transformation, promotes tumorigenesis, enhances tumor-associated angiogenesis. These carcinogenesis-related features of PTTG1 are likely attributable to its functions in mitosis, apoptosis, DNA repair and genetic modification. Hence, PTTG1 is regarded as an oncogene for pituitary tumors and other neoplasia.

Although the role of PTTG1 in tumor has been extensively studied, the regulation of PTTG1 remains poorly understood. A very recent study has shown that PTTG1 appeared to be regulated by its pseudogene PTTG3P in HCC [14]. Located at chromosome 8q13.1, although PTTG3P was regarded as a protein-coding gene, it is now realized that PTTG3P is a pseudogene, which shares similar structure with the parental genes that encode functional proteins, but contains some defects that prevent them from encoding fully functional proteins [28]. In this study, the authors elegantly showed that PTTG3P promotes HCC cell growth and metastasis via up-regulating PTTG1 and activating PI3K/AKT signaling [14]. Inspired by the exciting findings in this study, we aimed to figure out whether PTTG3P may have a similar function in CC, since our data from the CC patients showed increases in expression of both PTTG1 and PTTG3P, which were also positively correlated.

The effects of pseudogenes are complicated. At least, transcribing of the pseudogenes may provide a rich source of substrates for the post-transcriptional control of the parental genes, due to the highly homology of the sequence [28]. Indeed, this is why in most occasions, pseudogenes are found to be regulators of the parental gene levels. Here, we found that PTTG1 were increased by PTTG3P overexpression, but was inhibited by PTTG3P depletion in CC cells. However, PTTG3P levels were not altered by modulation of PTTG1 in CC cells, suggesting that PTTG3P is upstream of PTTG1, consistent with the general notion of the effects of a pseudogene. PTTG1 has been shown to be regulated by a number of microRNAs [7,29–31]. Therefore, it is expected that the alteration in PTTG3P may affect the PTTG1 levels through the competitive binding to the PTTG1-microRNAs.

CCNB1 is a cell cycle stimulator in G2-M phase transition. The activation of CCNB1 is regulated by CDK1, through phosphorylation of CCNB1 in the CCNB1-CDK1 complex. Once activated, CCNB1-CDK1 complex promotes different events of mitosis, including chromosome condensation, nuclear envelope breakdown, and spindle pole assembly. The open questions are: whether the action of CCNB1 here is conducted through PTTG1? Whether this process may be conducted through CDK1? PI3k/Akt signaling is involved in both the activation of PTTG3P [14] and the regulation of CCNB1 [32–34], suggesting a possible regulatory signaling pathway. In future, the effects of PTTG3P on CCNB1 should be further studied.

SNAIL is a very known factor that promote cell migration. E-Cadherin is very important for cells to maintain epithelial phenotype. During epithelial-mesenchymal transition, a process critical for cell migration, SNAIL and other factors may be activated, which subsequently inhibited E-Cadherin. Here, we detected upregulation of SNAIL and downregulation of E-Cadherin by PTTG3P, which support a role of PTTG3P in enhancing cell invasiveness. Again, the exact molecular mechanism may be further investigated in future studies.

The current therapy for CC includes surgery, radiotherapy and chemotherapy. Moreover, targeted therapy and immunotherapy have used Bevacizumab to suppress CC-associated angiogenesis, and have used Pembrolizumab to suppress PD-1, a protein on T cells to prevent their activation to attack tumor cells, respectively [20]. The current study highlights a novel molecular signaling pathway (PTTG3P/PTTG1) in control of growth and metastasis of CC, and sheds light on PTTG3P as a novel therapeutic target for CC.

## MATERIALS AND METHODS

### Ethic issue and patient specimens

All experimental protocols were approved by the Research Bureau of Xinxiang City Central Hospital. A total of 10 CC patients (aged from 36 to 42 years old, age has no effects on the results) were included in the study and followed up to five years. Written informed consent for the biological studies was obtained from each patient involved in the study. The specimens included the resected CC tissue and the paired adjacent normal cervical tissue (NCT). RT-qPCR for PTTG1 and PTTG3P was performed. The clinicopathological parameters were obtained histologically and clinically at Xinxiang City Central Hospital from 2011 to 2017.

### Cell Line culture and transfection

Human CC cell lines CaSki and HTB31 were included in the in vitro study. CaSki is an epidermoid cervical cancer cell line prepared by R.A. Pattillo in 1977 [35] and HTB31 is also called C33-A, prepared from a retinoblastoma in cervix from a 66-year-old female by N. Auersperg [36]. Both CC lines were purchased from American Type Culture Collection (ATCC, Rockville, MD, USA), and were maintained in Dulbecco’s modified Eagle’s medium (DMEM, Gibco, Gaithersburg, MD, USA) supplemented with 10% fetal bovine serum (FBS; Sigma-Aldrich, St Louis, MO, USA) in a humidified chamber at 37 °C in an atmosphere of 5% CO_2_. The plasmids of expression of transgene or shRNA for PTTG1 and PTTG3P under a cytomegalovirus (CMV) promoter were purchased from GeneCopoeia (Rockville, MD, USA). Transfection was done using 2µg plasmids by Lipofectamine 3000, according to the manufacturer’s instructions (Invitrogen, Rockville, MD, USA).

### CCK-8 assay

Cell counting kit-8 (CCK-8) assay, as performed to determine the viable cells, was applied using an CCK-8 kit, according to the manufacturer’s instructions (96992-500TESTS-F, Sigma-Aldrich). Briefly, a total of 5000 cells were seeded in each well of 24-well plates. The absorbance at 450 nm at 0, 24, 48 and 72 hours after cell seeding was measured.

### Transwell cell migration assay

Transwell cell migration assay was applied using a cell migration assay kit, according to the manufacturer’s instructions (ECM508, Sigma-Aldrich). Briefly, CC cells were seeded in the top chamber of a 24-well transwell insert (Millipore, Bedford, MA, USA) in serum-free media. The lower chamber was filled with media supplemented with 10% FBS. The cells that had migrated to the lower surface in 48 hours were fixed and subsequently stained with crystal violet for quantification.

### Analysis of cell proliferation

Cell proliferation was analyzed by 5-Bromo-2´-Deoxyuridine (BrdU)+ cells on total cells, using a BrdU in-situ detection kit (Becton-Dickinson Biosciences, San Jose, CA, USA).

### Analysis of cell apoptosis

The terminal deoxynucleotidyl transferase (TdT)-mediated dUTP nick-end labeling (TUNEL) was performed using a cy3-TUNEL staining kit (R&D Systems, Los Angeles, CA, USA). The positive controls used 10 minutes’ incubation at room temperature with 1500 U/ml DNAse1 in 50 mM Tris pH 7.5, 10 mM MgCl_2_ and 1 mg/ml BSA.

### RNA extraction, reverse transcription and quantitative RT-PCR (RT-qPCR)

Total RNA was extracted from the resected specimens from the patients or from the cultured cells with RNeasy kit (Qiagen, Shanghai, China). Complementary DNA (cDNA) was prepared from total RNA by reverse transcription using Omniscript reverse transcription kit (Qiagen). RT-qPCR was performed using the Quantitect SyBr green PCR system (Qiagen), and run in duplicates. All primers were purchased from Qiagen. Data were collected and analyzed using 2-△△Ct method for quantification of the relative mRNA levels. Gene expression levels were obtained through sequential normalization of the values to GAPDH and the experimental controls.

### Western blot

Total Protein was extracted from the cultured cells using RIPA buffer (Sigma-Aldrich). Equal amount of proteins was loaded in the gel. Primary antibodies for Western Blot are rabbit anti-CCNB1, anti-SNAIL, anti-E-cadherin and anti-GAPDH (Cell Signaling, St Jose, LA, USA). Secondary antibody is HRP-conjugated anti-rabbit (Jackson ImmunoResearch Labs, West Grove, PA, USA). Immunoreactivity was detected using chemiluminescence method (Thermo Scientific, San Jose, CA, USA). Images shown in the figure were representatives from 5 repeats.

### Statistical analysis

GraphPad Prism 7 (GraphPad, Chicago, IL, USA) software was used analyze data in the current study. Statistics on clinical specimens were done suing Wilcoxon Test, while data from in vitro studies were statistically analyzed using one-way ANOVA with a Bonferroni correction, followed by Fisher’s Exact Test for comparison between two groups. All values are depicted as mean ± standard deviation and are considered significant if p < 0.05. Bivariate correlations were calculated by Spearman's rank correlation coefficients. Kaplan-Meier curve was applied to record the overall survival of the patients included in this study.
